# Sister Mary Joseph Nodule as a Cutaneous Manifestation of Metastatic Appendiceal Adenocarcinoma: Case Report and Literature Review

**DOI:** 10.7759/cureus.2244

**Published:** 2018-02-28

**Authors:** Raghavendra L Girijala, Ryan R Riahi, Philip R Cohen

**Affiliations:** 1 College of Medicine, Texas A&M; 2 Dermatology, Derm Surgery Associates, PA; 3 Department of Dermatology, University of California, San Diego

**Keywords:** appendix, appendiceal, carcinoma, cutaneous, joseph, mary, metastasis, mucinous, nodule, sister

## Abstract

Sister Mary Joseph nodule (SMJN) is an uncommon pattern of superficial periumbilical tumor metastasis, with the primary tumor most commonly associated with gynecological or gastrointestinal origins. This manifestation can represent extensive tumor development from any of the intra-abdominal or pelvic structures. Therefore, SMJN carries a poor prognosis, with a two-year survival rate of only 13.5 percent regardless of the etiology of primary cancer. In this case, a 67-year-old man with metastatic mucinous adenocarcinoma of the appendix involving the umbilicus presenting more than five years after the initial cancer diagnosis is reported. The features of patients with metastatic appendiceal carcinoma presenting as SMJN are also reviewed. With the inclusion of our patient, there are six patients who have documented SMJN due to appendiceal carcinoma: two men, two women, and two patients without demographic data. The patients ranged from the ages of 31 to 68 years, with a median age of 56.5 years at cancer diagnosis and 59 years at SMJN diagnosis. In 75 percent of the cases, SMJN was the initial clinical manifestation of a previously unsuspected appendiceal carcinoma and presented clinically one to seven months (median of five months) before the pathologic confirmation of the metastatic appendiceal carcinoma. The likelihood of SMJN presenting as the initial clinical feature of appendiceal cancer may increase in patients with extensive intraperitoneal metastasis in the form of pseudomyxoma peritonei or carcinomatosis. Therefore, the observation of a solitary umbilical nodule should prompt an investigation for an underlying primary neoplasm, as the prognosis after tumor metastasis to the umbilicus is poor.

## Introduction

Sister Mary Joseph nodule (SMJN) refers to a morphologic presentation of cutaneous metastasis in which the tumor presents as a nodule in the umbilical region; it has classically been associated with adenocarcinomas of gastrointestinal origin. A 67-year-old man with SMJN and pseudomyxoma peritonei resulting from primary mucinous adenocarcinoma of the appendix is described. The features of patients with SMJN secondary to appendiceal carcinoma are reviewed.

## Case presentation

A 67-year-old man presented to his dermatologist for a total body skin check. He mentioned that he had an asymptomatic, but disfiguring, mass of two weeks duration that had developed in his umbilical region. He had previously been diagnosed with mucinous appendiceal adenocarcinoma 5.4 years earlier and was initially treated with hemicolectomy and systemic chemotherapy. The treatment resulted in partial tumor remission, and he was followed closely for the next three years.

Approximately 2.4 years ago, his metastatic disease progressed. He was treated with cytoreductive surgery (including splenectomy, omentectomy, and stripping of the right diaphragm) and hyperthermic intraperitoneal chemotherapy. He again had a partial remission and was followed closely. Recently, he developed an umbilical nodule that concerned him; however, he has not developed any tumor-related symptoms.

**Figure 1:**
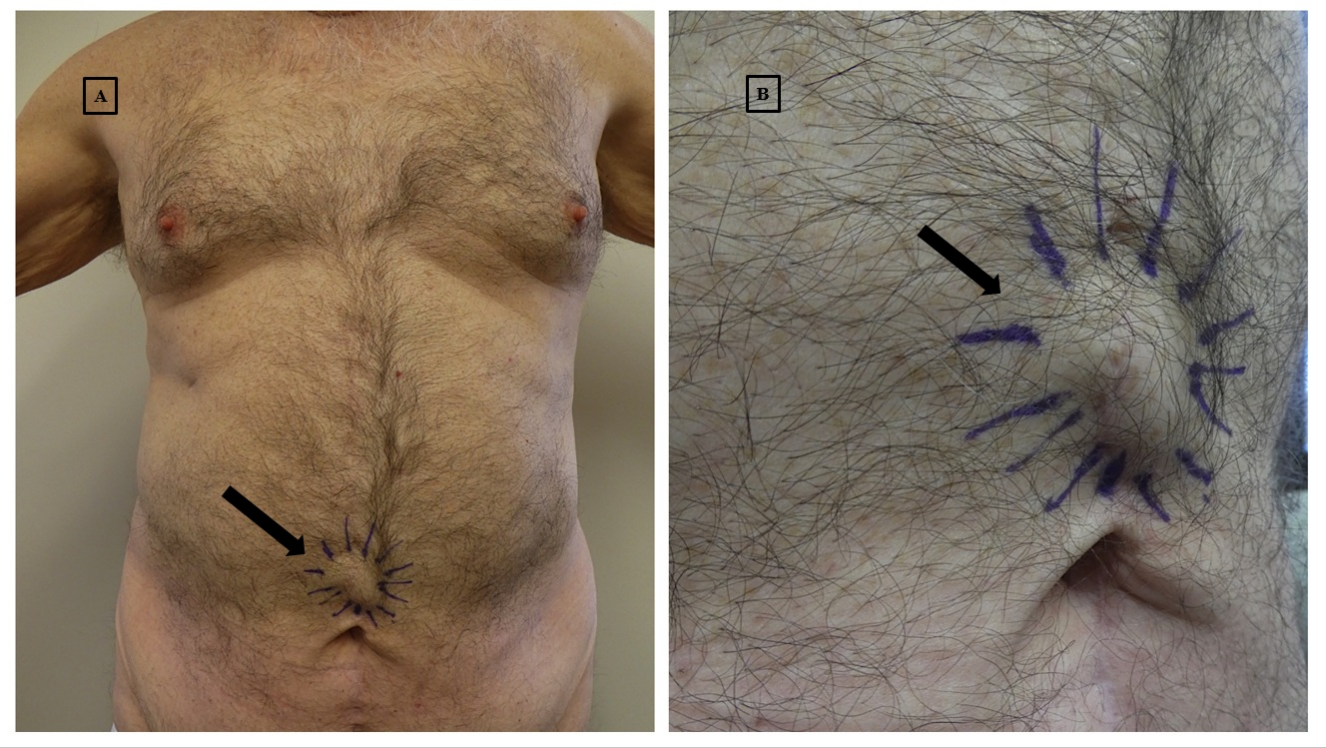
Sister Mary Joseph nodule secondary to an appendiceal mucinous adenocarcinoma Distant (a) and closer (b) views of a 3.5 x 3.0 cm Sister Mary Joseph nodule -outlined in purple ink (arrow) - presenting in the umbilical region with extension into underlying subcutaneous structures in a 67-year-old man with an established diagnosis of metastatic mucinous adenocarcinoma of the appendix.

A cutaneous examination demonstrated a 3.5 x 3.0 cm firm, non-tender, subcutaneous nodule on the abdomen in the umbilical region (Figure 1). On palpation, the insertion of the index finger in the umbilicus during the physical examination was blocked by the extension of the tumor. The tumor distribution was akin to an iceberg pattern, with a significant extension of the mass below the skin surface.

A computerized tomography (CT) scan of the abdomen and pelvis with intravenous contrast demonstrated a periumbilical subcutaneous nodule, peritoneal carcinomatosis, and malignant ascites consistent with pseudomyxoma peritonei. The physical exam in conjunction with radiographic findings established the diagnosis of SMJN secondary to mucinous adenocarcinoma of the appendix.

The patient is currently asymptomatic. Therefore, since there was neither perforation nor obstruction of his intestines, there was no need for an intervening surgery. After careful evaluation, his surgical oncologist elected for a close periodic follow-up until symptoms arise, including but not limited to, pain, bowel obstruction, or weight loss. Recommendations for follow-up include a computerized tomography scan of the abdomen and pelvis in six months.

## Discussion

Cutaneous metastases from internal malignancy are uncommon findings. In a study of 7316 cancer patients, only five percent of all patients developed cutaneous involvement over 10 years [1]. In another investigation of 4020 patients with known metastatic disease, only 10 percent manifested skin metastases over 10 years [2].

The identification of cutaneous metastases heralds a poor prognosis, with a life expectancy of one to 34 months. Cutaneous metastases can appear in any region of the skin. Morphologically, cutaneous manifestations can present as individual or grouped nodules, papules, telangiectasias, erythema, alopecia, and hyperkeratotic plaques. In a previously cancer-free individual, the discovery of visceral malignancy that has spread to the skin should prompt an evaluation for an underlying primary neoplasm [2-3].

Sister Mary Joseph nodule is a pattern of metastases referring to a firm nodule in the umbilical region. Sister Mary Joseph Dempsey, the surgical assistant of Dr. William Mayo, first observed this pattern of presentation. She was posthumously recognized for her contributions by Dr. Hamilton Bailey, who coined the term in 1949 [4].

Gastric, ovarian, and colorectal cancers are the most common primary tumors in patients with SMJN [1,4-5]. In contrast, SMJN has not been frequently documented secondary to appendiceal carcinoma. Including our patient, only six cases of SMJN secondary to appendiceal tumors have been reported (Table 1) [5-8].

**Table 1:** Characteristics of six patients with metastatic appendiceal carcinoma presenting as SMJN Abbreviations: ANLMP, appendiceal neoplasm of low malignant potential; As, Asian; C, case; Ca, Caucasian; CR, current report; CRC/HIPEC, cytoreductive surgery and hyperthermic intraperitoneal chemotherapy; Fo, following (months after); M, man; MA, mucinous adenocarcinoma; mo, months; NS, not stated; Pr, preceding (months prior); Ref, reference; SMJN, Sister Mary Joseph nodule; W, woman; y, years. ^a ^Cases 5 and 6 are of individuals who had appendiceal neoplasm of either low malignant potential or mucinous adenocarcinoma associated with SMJN; however, no additional details are provided by the investigators. ^b ^This is the age that their cancer was diagnosed. It is the same age as the diagnosis of SMJN in cases 1, 2, and 4; SMJN was diagnosed at age 67 years for case 3. ^c^ In cases 1, 2, and 4, the clinical presentation of an SMJN was observed seven, one, and five months (respectively) prior to the pathologic confirmation of metastatic appendiceal carcinoma.

C ^a^	Age (y)^ b^, Race, Gender	SMJN Onset ^c^	Tumor Subtype	Treatment	Follow-Up	Ref
1	31, Ca, M	Pr (7 mo)	MA	Ileocecal resection, omentectomy, and heated mitomycin-C infusion	Palliative care and death in two years	[6]
2	51, As, W	Pr (1 mo)	MA	Right colectomy, hysterectomy, bilateral salpingo-oophorectomy with refusal of adjuvant treatment	Patient lost to follow-up	[7]
3	62, Ca, M	Fo (65 mo)	MA	Hemicolectomy, systemic chemotherapy on first presentation, CRC/HIPEC for peritoneal metastasis	Periodic follow-up and imaging	CR
4	68, NS, W	Pr (5 mo)	MA	Abdominal surgery	NS	[8]

Of the six patients with appendiceal cancer-related SMJN, only four of the individuals have documented demographic, treatment, and follow-up data. Two of the patients were men and two of the patients were women. The median age for their primary cancer diagnosis was 56.5 years; their ages ranged from 31 to 68 years. However, the median age at SMJN diagnosis was 59 years; the men ranged in age from 31 to 67 years, while the women ranged in age from 51 to 68 years.

The discovery of SMJN preceded the diagnosis of appendiceal carcinoma in 75 percent of the patients. Specifically, in patients with appendiceal carcinoma-associated SMJN, the umbilical lesion (either a nodule, draining ulcer, or induration that was not suspected to represent metastatic cancer) clinically presented one to seven months (median of five months) before the diagnosis of metastatic appendiceal carcinoma was established. This is in contrast to the study by Lookingbill et al., which ascertained that only 0.8 percent of 7316 patients had skin involvement as the presenting sign [1].

Similar to our observation that SMJN often preceded the discovery of a disseminated appendiceal tumor, several investigators have also noted that SMJN was the initial of a previously unsuspected metastatic cancer; this includes ovarian, endometrial, cervical, gastric, small bowel, colon, and other tumors. Dubreuil et al., who conducted a literature review of 368 cases of SMJN between 1960 and 1995, noted that many of the patients (41.3 percent) had umbilical metastases discovered before the primary cancer; these tumors originated from locations such as the stomach, rectum, colon, small bowel, pancreas, ovary, and endometrium [4]. In addition, Papalas et al. reviewed 77 cases of SMJN at Duke University Medical Center from 1988 to 2011 and determined that 13 percent of cases presented with SMJN as the initial sign of a previously undiagnosed cancer [5]. Therefore, SMJN can often be the presenting sign of an unsuspected internal malignancy.

The diagnosis of SMJN can be determined by fine needle aspiration of the nodule and a histological analysis to ascertain the location of the primary tumor [4]. In patients with a history of an established solitary tumor, a radiographic analysis can be used to establish the diagnosis. The etiology of our patient’s SMJN was confirmed by a computerized tomography scan.

Appendiceal malignancy is a rare cause of SMJN. Indeed, Papalas et al. observed that in 58 cases of SMJN for which a primary tumor was identified, only 3.4 percent were due to appendiceal malignancy [5]. With regards to pathologic subtype, five of the six (83 percent) appendiceal cancer-associated SMJNs were in patients who had mucinous adenocarcinoma of the appendix [5-8]; one patient had an appendiceal tumor of low malignant potential [5].

The pathogenesis of mucinous appendiceal cancer presenting with umbilical metastasis as the initial clinical sign of disseminated malignancy is likely to be multifactorial. First, unlike other tumors, appendiceal cancer can be asymptomatic, presenting incidentally on colonoscopy, peri-operatively, or during imaging for other indications. Second, mucinous adenocarcinoma of the appendix often presents after the primary tumor has experienced micro- or macro-rupture, resulting in an abdominal collection of mucinous ascites known as pseudomyxoma peritonei. Furthermore, peritoneal carcinomatosis, as a manifestation of tumor metastasis, also occurs in appendiceal cancer, most commonly through intraperitoneal seeding and direct invasion. All of the patients with mucinous appendiceal adenocarcinoma-associated SMJNs had intraperitoneal dissemination. Therefore, it is plausible that patients who develop intraperitoneal metastasis in the form of ascites or carcinomatosis have an increased risk of presenting with SMJN before other systemic symptoms prompt clinical evaluation [9].

Metastasis involves the loss of local tumor cell adhesion and travel to regional or distant locations. Separation from local tissue occurs through the decreased levels or loss of function of adhesion molecules. Subsequently, cells invade the surrounding interstitial tissue via the upregulation of motility factors and degradative enzymes. Metastasis is ultimately achieved through direct extension, access to venous or arterial channels, or the lymphatic system [3].

Umbilical skin is unique in its anatomical proximity to intra-abdominal and pelvic malignancies and its connection to venous and lymphatic systems. As a result, metastasis can occur directly or indirectly. Contiguous extension is one postulated mechanism due to the umbilical tissue’s anatomical relationship with the anterior peritoneal tissue. Furthermore, numerous venous channels on the anterior abdominal wall, which vary by patient, involve the umbilicus. Finally, the umbilicus is a point of lymphatic intersection due to its access to the deep lymph system, such as para-aortic, internal mammary, external iliac nodes, and superficial nodes, including the axillary and inguinal nodes [4-5].

Regardless of tumor etiology, SMJN typically presents as a firm exophytic nodule varying in size from 0.5 to 15 cm, extending from the umbilicus above the abdomen; it may be painful [4,10]. Our patient’s metastatic tumor originated beneath and within his umbilicus and the surrounding subcutaneous tissue and extended upward as an abdominal mass. Indeed, his clinically visible mass was slightly superior to the umbilicus and had a morphology similar to that of an iceberg. Furthermore, the extension into underlying tissue was significant enough to block the insertion of the examiner’s index finger during the clinical examination.

Other atypical presentations of SMJN include ulceration with purulent, serous, or bloody exudate. Furthermore, SMJN can present as diffuse subcutaneous induration. Unless the possibility of a cutaneous metastasis is considered, SMJN is often misdiagnosed as a non-malignant lesion, such as an umbilical hernia. Therefore, the number of SMJN cases may be greater than what is represented in the literature [4].

The differential diagnosis of an umbilical nodule is broad and includes several benign conditions. In women of reproductive age, extra-uterine endometriosis must be considered; it can be differentiated by its variation in size and potential bleeding during menses. Umbilical or paraumbilical hernias are common; they can either be congenital or acquired after abdominal surgery. The differentiation of an umbilical hernia from SMJN is based on reducibility and associated cough impulse. Keloids are a potential cause of nodules in patients with a history of skin trauma or navel piercing. Other benign causes of umbilical nodules include epidermal inclusion cysts, fibromas, foreign body reactions, and pilonidal sinus [4,10].

Notably, in two of the three patients with appendiceal carcinoma-related SMJN that presented as the initial finding, the umbilical lesion was misdiagnosed as either eczema with secondary infection or as a sebaceous cyst [6-7]. In the patient reported by Srinivasaiah et al., the diagnosis of the primary appendiceal tumor was also delayed due to his prior open appendectomy; however, it was later noted that the appendiceal stump had been retained [6].

In contrast, primary umbilical malignancy is very rare. Clinically, they cannot be differentiated from metastatic internal tumors. A histological analysis by an experienced pathologist may yield insight into the location of a potential primary tumor [4,10].

The majority of SMJN are adenocarcinomas; however, undifferentiated carcinomas, carcinoids, leiomyosarcomas, and small cell carcinomas of the lung have also been noted, highlighting the range of tumors capable of disseminating to the umbilical region [4]. Mucinous appendiceal adenocarcinoma demonstrates glandular mucin-producing cells with or without signet ring cell components; these features can be observed in the metastatic site [9]. If an internal cancer is not detected after an extensive workup in a patient in whom an SMJN is suspected, the possibility of a primary neoplasm of the umbilicus should be considered.

Mucinous appendiceal adenocarcinoma management depends on the clinical presentation (ruptured versus non-ruptured appendix) and histological grade of the tumor. In low-grade, non-ruptured tumors, cautious appendectomy is recommended to prevent mucin spillage and potential seeding. In patients with non-ruptured, high-grade cancer, right hemicolectomy is conducted due to the higher risk of lymph node involvement [9].

Our patient underwent extensive treatment over five years. Given the initial presentation of a non-ruptured, high-grade tumor, he underwent right hemicolectomy and systemic chemotherapy. His subsequent peritoneal disease prompted cytoreductive surgery and hyperthermic intraperitoneal chemotherapy.

Patients with ruptured appendices can be classified as microscopic or gross peritoneal disease. For individuals with gross peritoneal disease, surgical cytoreduction (including total omentectomy, right lower quadrant peritonectomy, and right hemicolectomy) is performed to remove all gross disease, followed by a single dose of intraperitoneal chemotherapy with mitomycin C. Postmenopausal women are advised to undergo bilateral oophorectomy due to the ovaries being a common site of metastasis, whereas premenopausal women can retain their ovaries, given that they undergo routine follow-up with ultrasound [9].

Carcinoma of the appendix can initially present as appendicitis; this occurs when there is microscopic rupture of the appendix. Intra-operative pathology to ascertain the grade of the tumor is vital. Low-grade cancers are managed with a resection of the residual disease, sparing patients of intraperitoneal chemotherapy due to the low risk of recurrence. In contrast, high-grade tumors are treated similarly to gross peritoneal disease with cytoreductive surgery and intraperitoneal chemotherapy [9].

In patients with appendiceal mucinous adenocarcinoma, surgical and medical care (with cytoreduction and intraperitoneal chemotherapy) still often result in recurrence. In low-grade and high-grade appendiceal mucinous tumors, the median disease-free survival is 38 and 21.6 months, respectively. For these individuals, repeat debulking and intraperitoneal chemotherapy are potential options [9].

The observation of SMJN is an ominous sign. Dubreuil et al. noted that once SMJN is diagnosed, the survival time is an average of 11 months, with only 13.5 percent of patients living after two years, regardless of the primary tumor etiology [4]. The average survival time for patients after the diagnosis of SMJN associated with appendiceal mucinous adenocarcinomas remains to be determined; one man survived for two years and our patient is currently alive a few months after the detection of his umbilical metastasis.

## Conclusions

SMJN is a cutaneous metastasis in which the metastatic tumor presents as an umbilical mass. Appendiceal cancer-related SMJN has only been described in six individuals, including our patient: two men, two women, and two individuals for whom demographic information was not reported. The median age of their primary cancer diagnosis was 56.5 years whereas the median age of the detection of their SMJN was 59 years. In 75 percent of the patients, SMJN was the initial clinical feature of a previously unsuspected malignancy; it was clinically noted one to seven months (median of five months) prior to the pathologic confirmation of the metastatic appendiceal malignancy. Prognosis is significantly affected by the discovery of an SMJN, highlighting the need for an appropriate workup, possibly including biopsy, in an individual upon the detection of a new umbilical nodule.
